# Georgia Surgeons’ Club

**Published:** 1913-12

**Authors:** Geo. H. Noble

**Affiliations:** Gynecological Clinic at the Grady Hospital, Atlanta, Ga.


### Journal-Record of Medicine

Successor to Atlanta Medical and Surgical Journal, Established 1855
and Southern Medical Record, Established 1870

**OWNED BY THE ATLANTA MEDICAL JOURNAL COMPANY**

PUBLISHED MONTHLY

*Official Organ Fulton County Medical Society, State Examining Board, Presbyterian Hospital, Atlanta, Birmingham and Atlantic Railroad Surgeons' Association, Chattahoochee Valley Medical and Surgical Association, Etc.*

EDGAR BALLENGER, M. D., Editor
BERNARD WOLFF, M. D., Supervising Editor
A. W. STIRLING, M. D., C. M., D. P. H.; J. S. HURT, B. Ph., M.D.
GEO. M. NILES, M. D., W. J. LOVE, M. D., (Ala.); Associate Editors
E. W. ALLEN, Business Manager

**COLLABORATORS**

DR. W. F. WESTMORLAND, General Surgery
F. W. McRAE, M. D., Abdominal Surgery
H. F. HARRIS, M. D., Pathology and Bacteriology
E. B. BLOCK, M. D., Diseases of the Nervous System
MICHAEL HOKE, M. D., Orthopedic Surgery
CYRUS W. STRICKLER, M. D., Legal Medicine and Medical Legislation
E. C. DAVIS, A. B., M. D., Obstetrics
E. G. JONES, A. B., M. D., Gynecology
R. T. DORSEY, JR., B. S., M. D., Medicine
L. M. GAINES, A. B., M. D., Internal Medicine
GEO. C. MIZELL, M. D., Diseases of the Stomach and Intestines
L. B. CLARKE, M. D., Pediatrics
EDGAR PAULIN, M. D., Opsonic Medicine
THEODORE TOEPEL, M. D., Mechano Therapy
R. R. DALY, M. D., Medical Society
A. W. STIRLING, M. D., Etc., Diseases of the Eye, Ear, Nose and Throat
BERNARD WOLFF, M. D., Diseases of the Skin
E. G. BALLENGER, M. D., Diseases of the Genito-Urinary Organs

VOL. LX. ATLANTA, GA., DECEMBER, 1913. NO. 9

### GEORGIA SURGEONS' CLUB.

DR. GEO. H. NOBLE,
GYNECOLOGICAL CLINIC AT THE GRADY HOSPITAL,
ATLANTA, GA.

Dr. Geo. H. Noble, Gynecological Clinic at the Grady Hospital, Atlanta, Ga.

Case No. 1. Lacerated perineum; retroverted uterus.

The cervix was dilated and the uterus curetted. The perineum was repaire dby denudation of scar tissue, isolation and end-to-end union of the separated levators, and suturing face-to-face separately the strong structures of the perineum, namely, the recto-vesicle fascia an the triangular ligament, and by completing the closure with a purse string suture through these structures and including the reflection of fascia over the levator and muscle.

Suspension of the uterus was done by drawing loops of the round ligaments extraperitoneally through small incision in the aponeurosis anterior or the rectus muscle. The incision in the aponeurosis was then closed with kangaroo tendon. Method of closing the abdomen was also demonstrated.

Case II. Acute pelvic peritonitis; drainage. (Dr. Dean F. Winn):

This patient had been kept in the wards some time in order that she might recover as far as possible, through rest in bed, etc., from an acute double pyosalpinx. On the day before operation, after having had a normal temperature for several days, she developed diffuse pelvic peritonitis. Her general condition grew progressively worse so that abdominal drainage was deemed wise. Free pus was found in several pockets. Radical operation was not attempted.

Case III. Chronic colitis; ileo-sigmoid anastomosis:

The ileum was resected about two inches from the ileocecal junction by dividing the gut between clamps with an electric cautery. The severed ends were invaginated with purse-string sutures. Side-to-side anastomosis was then made between the proximal portion of the ileum and the lowest available point of the sigmoid. Intestinal loops were drawn through slits in a piece of sheet rubber which was large enough to protect the entire field of operation from contamination. The anastomotic opening was closed by suturing together the edges with a continuous, through and through No. 1 iodine catgut and reinforcing with a linen Lembert stitch.

Some difficulty was encountered in liberating the sigmoid sufficiently, it having become adherent to the cervical stump following a previous supravaginal hysterectomy. There were also old adhesions between the sigmoid and the left side of the pelvic wall. The portion of gut adherent to the cervix was somewhat browny as a result of a small adscess in the locality so that it was necessary to repair a slight rent in the gut made in separating the adhesion.

A long rectal tube was passed through the rectum, sigmoid, the anastomotic opening and into the small intestine.

A small rubber catheter was placed in the distal end of the ileum (the end of gut invaginated around it) and brought through a stab wound in the abdominal wall. The ileum and tube were fixed by sutures at this point.

Bismuth pictures were used to demonstrate the enteroptosis and dilated colon.

---

DR. EDGAR G. BALLENGER, CLINIC AT 11 A. M. WEDNESDAY AT THE ATLANTA MEDICAL COLLEGE, DEMONSTRATION OF INTRAVENOUS INJECTIONS OF SALVARSAN AND NEOSALVARSAN. The 50-c. c. syringe shown herewith facilitates the intravenous injections of salvarsan and neosalvarsan. For some time we had administered neosalvarsan with a 20-c. c. Leon syringe. We also cautiously administered medium size doses of salvarsan in this manner, but found that phlebitis would occasionally follow when the remedy was so slightly diluted. By diluting the salvarsan with 50-c. c. of sterile water, rendering the solution weakly alkaline, and injecting it slowly, allowing the blood flowing through the vein to provide further dilution, we have not had any untoward effects from the salvarsan. We think, dose for dose, salvarsan is more potent than neosalvarsan, though the latter is absorbed better if given intramuscularly, and so we always administer the neosalvarsan to babies where it must be given intramuscularly. The small caliber of the needle used, besides making its accurate introduction easy, makes it practically impossible to inject the solution too rapidly. The shoulder of the larger Leur and Record syringes interfered with the introduction of the needle and the injection of the solution. We devised, therefore, a syringe with the needle offset from the center so that the nedle may be easily introduced into the vein parallel with it. An ordinary ¾ inch 24-gauze needle may be used; the necessary pressure ininserting it does not flatten the vein and thus prevnts passing the needle through the posterior wall of the vein. Slight traction on the piston draws a few drops of blood into the barrel of the syringe and shows if the needle has entered the lumen of the vein. While the injection is being made the syringe lies flat upon the arm where it may be steadied. An ordinary soft rubber retention catheter wrapped twice around the arm and slightly tucked under will give satisfactory distention of the veins. The elasticity of the retention catheter is superior to the ordinary catheter or tourniquet and immediately unwraps itself when released, provided this part of the arm does not rest upon the table. In this manner the needle is prevented from being disturbed while releasing the tourniquet. The very small hole left by the needle soon heals without leaving any tell-tale marks or scars. About 0.3 to 0.4 of salvarsan or 0.4 to 0.6 of neosalvarsan may thus be administered to ambulant patients every one or two weeks, until seven, eight, or more injections have been administered, and more when necessary to secure permanently negative tests of the blood and spinal fluid. Mercury and iodides should also be given as indicated. Only slight reactions are produced by these small doses of salvarsan and neosavlarsan, and the patients do not object to prolonged courses of the remedies when so administered. A free supply of water should be drunk by the patient during the treatment, so as to dilute well the remedy during its elimination through the kidneys and thus prevent their irritation. The small doses frequently repeated effect a more satisfactory cure of syphilis and with less disturbance and danger than large doses administered at longer intervals. For less robust patients smaller doses should be administered and repeated about once a week until the desired result is obtained.

